# Process evaluation of knowledge transfer across industries: Leveraging Coca-Cola’s supply chain expertise for medicine availability in Tanzania

**DOI:** 10.1371/journal.pone.0186832

**Published:** 2017-11-09

**Authors:** Erika Linnander, Christina T. Yuan, Shirin Ahmed, Emily Cherlin, Kristina Talbert-Slagle, Leslie A. Curry

**Affiliations:** 1 Yale Global Health Leadership Institute, New Haven, Connecticut, United States of America; 2 Yale School of Public Health, New Haven, Connecticut, United States of America; 3 Yale School of Medicine, New Haven, Connecticut, United States of America; Perdue University, UNITED STATES

## Abstract

Persistent gaps in the availability of essential medicines have slowed the achievement of global health targets. Despite the supply chain knowledge and expertise that ministries of health might glean from other industries, limited empirical research has examined the process of knowledge transfer from other industries into global public health. We examined a partnership designed to improve the availability of medical supplies in Tanzania by transferring knowledge from The Coca-Cola system to Tanzania’s Medical Stores Department (MSD). We conducted a process evaluation including in-depth interviews with 70 participants between July 2011 and May 2014, corresponding to each phase of the partnership, with focus on challenges and strategies to address them, as well as benefits perceived by partners. Partners faced challenges in (1) identifying relevant knowledge to transfer, (2) translating operational solutions from Coca-Cola to MSD, and (3) maintaining momentum between project phases. Strategies to respond to these challenges emerged through real-time problem solving and included (1) leveraging the receptivity of MSD leadership, (2) engaging a boundary spanner to identify knowledge to transfer, (3) promoting local recognition of commonalities across industries, (4) engaging external technical experts to manage translation activities, (5) developing tools with visible benefits for MSD, (6) investing in local relationships, and (7) providing time and space for the partnership model to evolve. Benefits of the partnership perceived by MSD staff included enhanced collaboration and communication, more proactive orientations in managing operations, and greater attention to performance management. Benefits perceived by Coca-Cola staff included strengthened knowledge transfer capability and enhanced job satisfaction. Linking theoretical constructs with practical experiences from the field, we highlight the challenges, emergent strategies, and perceived benefits of a partnership across industry boundaries that may be useful to others seeking to promote the transfer of knowledge to improve global health.

## Introduction

Persistent gaps in the availability of essential medicines are a rate-limiting barrier toward the achievement of global health targets [1–4]. Although stockouts occur in high-income countries [5–8], the most critical gaps have been observed in low-income countries, where estimates suggest that fewer than half of children receive essential medications, and only a third of women receive medicines to manage major causes of maternal death [9]. Supply chain bottlenecks result in stock-outs of priority commodities in over 40% of health facilities in sub-Saharan Africa [10–13].

A variety of approaches have been developed to improve medicines availability [14–19]; however, as critical gaps persist, the global health community is looking across industry and sector boundaries to seek global supply chain solutions [20] from multi-national corporations outside of health. Knowledge transfer is the process through which organizations capture, collect and share knowledge, so that it can be accessed and used by others [21, 22], including actors from other industries [23, 24]. Despite the wealth of knowledge that global health organizations might glean from other industries, transferring knowledge across organizational boundaries [22, 25–29] remains challenging. Although prior work provides theoretical insight into knowledge transfer [30], few empirical studies have explored concrete challenges to knowledge transfer across different industries [31], or identified approaches to address such challenges during partnership implementation, particularly within the realm of global health.

In this paper, we explore knowledge transfer across industry (i.e., medical and beverage) and sector (i.e., public and private) boundaries. We describe a collaboration whose partners include Coca-Cola, a private beverage system comprised of The Coca-Cola Company (TCCC) and its bottlers, and Tanzania’s Medical Stores Department (MSD), an autonomous department of Tanzania’s Ministry of Health and Social Welfare responsible for the procurement, storage, and distribution of essential drugs and medical supplies for the country. The aim of this process evaluation [32, 33] was to document the experience of knowledge transfer across industry boundaries, including challenges and approaches to address those challenges, from the perspective of the primary partners. Findings can help inform implementation of similar cross-industry partnerships to address key challenges in global health.

### Partnership setting

A perennial question in the global health community has been: if one can find a bottle of Coca-Cola in many of the world’s most hard to reach locations, then why can’t one find life-saving drugs and medical supplies? In 2009, executives from The Coca-Cola Company, The Global Fund to Fight AIDS, Tuberculosis and Malaria (The Global Fund), and the Bill & Melinda Gates Foundation (The Gates Foundation) envisioned a novel response: to transfer Coca-Cola’s global supply chain expertise to the public health sector.

In 2010, with local support from Accenture Development Partnerships (ADP), and Coca-Cola Kwanza (CCK) (a Coca-Cola bottling company in Tanzania), the partners (Table 1) launched an effort to improve medicines availability in Tanzania by transferring Coca-Cola’s supply chain expertise to MSD [12]. From 2010–2013, implementation occurred in four phases, with a focus on three workstreams: (1) Last mile logistics (to optimize distribution routes); (2) Core planning and procurement (to support more proactive procurement); and (3) Talent management (to fill human resource gaps). Additional detail on design and implementation can be found in the online case study [34]. In 2011, Yale University received funding from TCCC to conduct a process evaluation of the partnership.

**Table 1:** Partner organizations.

Organization Name	Organization Description	Role in the Partnership
The Coca-Cola Company (TCCC)	Multinational beverage corporation and manufacturer, retailer and marketer of nonalcoholic beverages. Serving 1.7 billion consumers a day in more than 200 countries, the production and distribution of Coca-Cola follows a franchising model in which TCCC provides a concentrate to its bottling partners who then manufacture, package, distribute, and sell products for local consumption.	Private sector champion and knowledge donor: Donated core business expertise on supply chain management to MSD; seconded a dedicated project lead from the Coca-Cola system; funded Yale process evaluation.
Coca-Cola Kwanza (CCK)	One of three Coca-Cola bottling partners in Tanzania (Coca-Cola Kwanza, Nyanza Bottling Co. Ltd., and Bonite Bottlers Limited), responsible for manufacturing beverages and distributing final products to consumers in Tanzania.	Knowledge donor: Donated core business expertise on supply chain management to counterparts at MSD.
Medical Stores Department (MSD)	Autonomous department of the Ministry of Health and Social Welfare, responsible for procurement, storage, and distribution of essential drugs and other medical supplies in Tanzania.	Knowledge recipient: Incorporated knowledge transferred from The Coca-Cola Company and Coca-Cola Kwanza into MSD systems and processes.
Accenture Development Partnerships (ADP)	Non-profit affiliate of Accenture that aims to channel skills and resources of Accenture’s global management consulting talent to the international development sector.	Knowledge broker: Received funding to coordinate partnership implementation and facilitate packaging and translation of Coca-Cola’s core business expertise and processes to MSD.
The Global Fund to Fight Aids, TB & Malaria (The Global Fund)	International financing organization that aims to prevent and treat HIV and AIDS, tuberculosis and malaria.	Partnership sponsor: Jointly conceptualized partnership and engaged partners to address supply chain issues.
The Bill and Melinda Gates Foundation (the Gates Foundation)	Private grant-making foundation that aims to enhance healthcare and reduce global poverty.	Partnership sponsor: Jointly conceived of the partnership and catalyzed funding for ADP support.
Yale University	Research institute that develops leadership at Yale University and around the world through education and research programs aimed at strengthening health systems and promoting health equity and quality of care.	Process evaluator: Received funding to study partnership processes to inform implementation and replication of model in other settings.

## Methods

This process evaluation [35] included in-depth interviews with individuals from each of the partner organizations. We explored participant perceptions of the knowledge transfer process during multiple stages of the partnership. The study was developed in accordance with established standards for qualitative research as reflected in the COREQ guidelines [36].

### Sample

We used a purposeful sampling approach with snowball sampling [35] to identify individuals with an in-depth understanding of the partnership (or ‘key informants’) [35]. We recruited 2–3 individuals from each partner organization/role combination, for a total of 70 interviewees (Table 2).

**Table 2:** Count of in-depth interviews by partner organization.

	July–October, 2011	October, 2012	May, 2013	May, 2014	Total
The Coca-Cola Company (TCCC)	4	1	1	0	6
Coca-Cola Kwanza (CCK)	3	0	5	4	12
Medical Stores Department (MSD)	10	6	5	5	26
Accenture Development Partnerships (ADP)	10	4	0	1	15
The Global Fund to Fight Aids, TB & Malaria	3	2	0	0	5
The Bill and Melinda Gates Foundation	1	0	0	0	1
Other*	2	1	2	0	5
**Total Interviews**	**33**	**14**	**13**	**10**	**70**

*Other organizations included: John Snow, Inc. (JSI), the United States Agency for International Development (USAID), and Supply Chain Lab. Although not official partners, these organizations were engaged with MSD on parts of this and other projects to improve supply chain management in Tanzania.

### Data collection

We conducted four waves of interviews corresponding to phases in partnership design and implementation: July–October 2011 focused on partnership development with high-level officials, October 2012 and May 2013 focused on implementation with individuals directly involved in the knowledge transfer process, and May 2014 included repeat interviews with 10 informants from CCK, MSD and ADP to understand their experience with longer-term implementation and sustainability in the year after the project had closed. Interview waves were conducted by 3–4 experienced researchers from Yale representing diverse disciplines including public health, health services research, and health care management. Interviews were conducted in person in Tanzania, with the exception of several headquarter-based participants from TCCC, the Global Fund, the Gates Foundation, and ADP who were interviewed on the phone due to travel constraints.

The discussion guide focused on understanding perceptions of how the knowledge transfer partnership was developed and implemented, with probes for the types of knowledge exchanged, factors that enabled or hindered knowledge transfer, and perceived changes in organizational practice or culture. Interviews lasted approximately 45–60 minutes. After obtaining verbal consent from participants, interviews were audio-taped and professionally transcribed.

### Data analysis

We used an integrated approach to developing our code structure [35, 37, 38]. We began with an organizing framework based on Easterby-Smith, Lyles, and Tsang’s [28] conceptual framework of inter-organizational knowledge transfer and Szulanski’s [26] stages of the knowledge transfer process. We then developed the code structure inductively using the constant comparative method [38, 39]. The process of refining codes and describing properties of each continued until we achieved theoretical saturation [35, 39].

Four members of the research team each coded the data independently, iteratively developing, finalizing and applying the code sheet. Discrepancies in codes were resolved through negotiated consensus. Throughout coding and analysis, research team members were explicitly encouraged to challenge discrepant views, and the code sheet included codes for disconfirming information. [40–42] We used ATLAS.ti Scientific Software (Version 7.0).

### Ethics statement

Prior to the start of the study, a protocol application (Protocol #1106008643) was submitted and reviewed by the Yale University Human Research Protection Program (HRPP) Human Subjects Committee. The protocol was approved following expedited review by the Human Subjects Committee and was found to be of minimal risk to meet approval requirements under University IRB policy and 45 CFR 46 as applicable. All study participants provided their verbal informed consent to participate in this study. A copy of the manuscript was shared with a representative from each partner organization (Table 1) before submission for publication. Partners provided comments, but no changes were made to the findings reported in the manuscript.

Mindful of the potential conflicts of interest posed by the funding structure supporting this project, we used several established techniques for explicit critical reflection: 1) data collection and analysis were performed by a multidisciplinary team representing public health, anthropology, and health management, with members explicitly encouraged to challenge discrepant views and diverse perspectives [42]; 2) at the onset of the project, we discussed preconceptions and concerns regarding the inherent ethical challenges of working with Coca-Cola, as well as the potential for Coca-Cola to gain public and policy credibility through the project [43]; 3) we actively engaged in reflexivity to explore and challenge our preconceptions throughout data collection, analysis, and synthesis [44]; and 4) in the analysis phase, we systematically considered competing or alternative conclusions from the data [40].

## Results

### Challenges and emergent strategies

Partners identified several challenges during partnership implementation. Because the project was dynamic and unfolding in a complex environment, resulting strategies to address each challenge emerged through real-time problem solving (Table 3). Challenges and emergent strategies are described below, with illustrative quotations for each.

**Table 3:** Challenges and emergent strategies.

Challenges	Emergent strategies
Identifying relevant knowledge, expertise or practice to transfer	• Leverage receptivity of MSD leadership • Engage a boundary spanner to identify concrete knowledge to transfer • Promote local recognition of commonalities across industries
Creating operational solutions that were feasible for MSD	• Engage experts to manage translation activities • Develop tools with visible benefits for MSD
Maintaining momentum between project phases	• Invest in local relationships/roles • Provide time and space for the partnership model to evolve

#### Challenge #1: Identifying relevant knowledge, expertise or practice to transfer

In the initial phase, partners had difficulty articulating the concrete knowledge, expertise, or practice that Coca-Cola could transfer and that MSD would value. Trial and error was needed for MSD to articulate specific gaps in their operations that could be addressed through Coca-Cola’s core business expertise and for Coca-Cola to identify the specific processes and ways of working that matched the needs and local realities of MSD. Executives from Coca-Cola reflected on early struggles to define what new knowledge would be of value to MSD:

*It was a massive learning experience… we wanted to hear and understand what the needs were like before we put on the table what specifically we could offer*. *So it was a bit of a chicken and egg*. (ID3)

MSD staff also expressed initial skepticism about the relevance of Coca-Cola’s expertise to medicines and medical supplies. As one MSD employee reflected, the distribution of beverages to villages was a ‘completely different animal’:

The Global Fund came with Coca-Cola to MSD to tell us about partnership with Coke. The whole management team was just staring at them. What are these people talking about?…This is a completely different animal—Transporting a bottle of Coke to the village and transporting medicine to the village.… I mean you can't compare these two… (ID39)

#### Emergent strategies to address challenge #1

Three strategies emerged to help identify knowledge that could be translated from Coca-Cola to MSD: leveraging the receptivity of MSD leadership, engaging a boundary spanner to identify concrete knowledge to transfer, and promoting local recognition of commonalities across industries.

First, the partners leveraged receptivity of MSD leadership. Despite initial skepticism from both senior and mid-level MSD staff, the MSD Director General emerged as a champion of the project. He was receptive to non-traditional partnerships and recognized the potential for transferability of Coca-Cola’s knowledge. The Director General bridged cultural divides (a Tanzanian who had been educated in the United States) and industrial divides (experienced in several different industries) to bring the project to MSD senior management. This receptivity at the top was translated down the organizational hierarchy to those in critical operational roles, such as demand planners, logistics managers, and human resource managers. An MSD official recounted that once he was ‘sold’ on the issue, he worked towards convincing his peers of the potential merits of the partnership:

*I will share with you openly that not everybody*, *even in my management team*, *was sold on this issue …I was sold first and then I started convincing my fellow directors why this is important…As we started moving along*, *[ADP] came in and [we] saw how systematic and professional they were approaching the problems and proposing solutions*. *People can see the light bulbs going off*. *Okay*, *maybe this will lead to something*. (ID7)

Second, the partners engaged a boundary spanner to identify concrete knowledge to transfer. Boundary spanners are individuals who facilitate the sharing of expertise by linking two or more groups of people separated by location, hierarchy, or function [45]. To span organizational boundaries in the early stages, Coca-Cola contracted an expert who had extensive experience in Coca-Cola supply chains in Africa to conduct an analysis of MSD and Coca-Cola’s value chains. This expert had a “passion for development work” (ID3) and had worked with Coca-Cola on prior corporate social responsibility (CSR) initiatives. He was able to identify 13 concrete opportunities where Coca-Cola tools and expertise closely matched MSD needs. When describing the role of this consultant, one respondent emphasized the importance of identifying specific, clearly defined opportunities between partners:

We worked on the ground with a local Coca-Cola Kwanza bottling facility to better understand a few things…how they currently run their supply chain operation, looking at people, process and technology…what skills do they have, what training do they have in place in order to meet the [MSD] skills gap (ID33)

Third, the partners worked to promote local recognition of commonalities across industries. As the partners explored areas for collaboration, they visited each other’s warehouses and discussed shared challenges of operating in Tanzania, including difficult climate and road conditions, import and export controls, telecommunication gaps, and difficulties in recruiting skilled staff. These shared experiences created the opportunity for collaboration between mid-level/operational staff from each organization. As a staff member from Coca-Cola Kwanza reflected:

One of the things [MSD] learned was that the challenges that we face are the same challenges that they face…clearing containers from the port, transferring product around the country. So, it was actually an eye-opener to them that they are not unique in these challenges…I think the big learning was how we proactively plan to mitigate these challenges. (ID56)

#### Challenge #2 Creating operational solutions that were feasible for MSD

A second challenge was developing solutions that were feasible for implementation within MSD. Coca-Cola’s solutions had developed within their particular cultural and regulatory environment, and required adaption to fit MSD’s environment. For example, although Coca-Cola had extensive knowledge about how to respond to their market, they were not obligated to distribute to unprofitable locations. Consequently, Coca-Cola had limited knowledge about some of the most remote markets. Commenting on these differences, a USAID representative in Tanzania expressed:

While the supply chain has a lot of similarities between public sector and private sector, and there are a lot of core competencies that can be pulled out, they are inherently involved in serving completely different markets. The public sector has to go to places whether it makes business sense or not. Coke can choose not to go there. (ID57)

Furthermore, some of Coca-Cola’s operating models (such as performance based compensation and contracting out to distributors) posed a challenge for MSD to adopt due to government regulations. Although MSD enjoyed considerable autonomy, it required government approval for any major changes to its organizational structure. When Coca-Cola recommended a performance-based compensation structure, implementation within MSD was slowed by review for approvals from the Ministry and labor unions. Coca-Cola staff described the barriers:

They are different from us because we are a private sector. We have [more] autonomy [than they do] because they're under the Ministry of Health. It's very difficult for them…because they have to follow the bureaucratic chain to get it done. (ID52)

Similarly, although Coca-Cola shared extensive information regarding the use of third-party distributors, MSD had to overcome regulatory barriers to outsourcing. As one MSD employee commented:

We have this drug regulatory authority…who [is] responsible for regulating the business of pharmaceuticals in the country…It’s very easy for Coke to say, okay, now we want in this area distributors for Coca-Cola….With [medicine], you have to comply to the regulations. (ID13)

#### Emergent strategies to address challenge #2

Emergent strategies to adapt Coca-Cola solutions to MSD’s context included the engagement of experts to manage translation activities and the development of tools with visible benefits for MSD.

First, the partners engaged experts to manage translation activities. Neither Coca-Cola nor MSD was well positioned to translate Coca-Cola practices into MSD operations. To fill this role, Coca-Cola engaged consultants from ADP to manage day-to-day partnership operations and translate Coca-Cola’s knowledge for MSD. Working collaboratively, ADP adapted and packaged Coca-Cola knowledge, expertise and practices in a way that was useful and feasible for MSD. An ADP manager described:

You can have technical knowledge from a private sector organization, you can have the will from the recipient organization… but you need somebody to facilitate it all and make it happen…getting stakeholders together and coming with and working through solutions. I think it’s that glue in the middle. (ID27)

A respondent from CCK described the role of ADP:

ADP played a very big role because they broke it up into different work streams…I allocated each one of my functional aides into those work streams and ADP had a person heading up each of those work streams and I think that’s how the knowledge transferred. (ID10)

Second, the partners developed tools with visible benefits for MSD. ADP introduced a number of practical tools adapted from Coca-Cola to enhance skills of MSD staff [34]. ADP provided organized site visits to Coca-Cola’s bottling facilities to help MSD employees understand how Coca-Cola’s tools and procedures could be applied within MSD. ADP also used participatory knowledge transfer techniques to engage staff and teach practical aspects of business operations common in the private sector. For example, the “Supply Chain Game,” adapted from a game developed at MIT [46], was used to highlight the importance of planning and communication. MSD staff noted the value of this experience:

The game went on and people are noting, “So, I have a role to play in improving this performance.” …That thing brought us together, as an organization… It was difficult at first but through these games everybody in the organization understands that we really need to do it the way Coke does. (ID50)

#### Challenge #3: Stalled momentum between project phases

Because the partnership was a start-up venture, partners worked in discrete pilot phases to demonstrate proof of concept, punctuated by periods dedicated to parlaying early results into additional financial investment in the partnership. Momentum stalled between phases, as described by one respondent from Coca-Cola:

One of the limitations of this project has been the funding cycle. We’ve had too long a gap between phases driven by a lack of funding for ADP. We’re working so hard on a global collaborative agreement, where we have a multi-year funding mechanism that we can draw down on and therefore make quick decisions as a partnership group rather than have to apply on an individual basis for funding. But, look that’s a natural reality of starting something. You won’t have a multi-year funding mechanism up front. We haven’t proven anything. (ID 58)

Each phase had to overcome the inertia of these gaps, a process that was complicated by staffing changes and competing demands within each partner organizations.

Turnover within each organization limited continuity in key roles. There was no single “custodian” (ID 53) on the ground in Tanzania holding the partnership together between phases. Some turnover was planned (each phase was staffed by a different team of consultants from ADP) and some was driven by natural attrition of individuals. Describing turnover at CCK, one participant described:

Our challenge on this project is being that there’s been quite a high staff turnover in the time period that we’ve been working with them as a bottler. And so it definitely impacts our access because you start building a relationship with a new person and you build trust with a new person. (ID 4)

MSD and Coca-Cola staff needed to manage competing demands (MSD working on several large-scale reform efforts, and Coca-Cola balancing CSR with core business operations), and needed to be re-engaged with each phase. An MSD employee reflected on the challenges of prioritizing:

We were implementing several other projects. So, it was very difficult to prioritize….The same people are supposed to be in two different places at the same time. That was a big challenge, to align people. By the end of the day, we managed but we should say that some areas also suffered. (ID50)

#### Emergent strategies to address challenge #3

Two strategies emerged to address stalled momentum: investment in local relationships and roles, and provision of time and space for the partnership to evolve.

First, the partners investmented in local relationships and roles. The partners recognized that relationships between staff at CCK and MSD would be critical to ensuring the sustainability of the partnership beyond the phases that were actively supported by ADP. Counterparts at MSD and Coca-Cola were matched as connection points for each of the project’s workstreams. A staff member at CCK described the match between demand planners (individuals responsible for forecasting demand for products from outlying distributors or facilities):

“I expect our demand planner to keep visiting their demand planner and attend their meetings. But now it’s dependent on MSD. We won’t close our doors; we have told them. If they need us, they will get us.” (ID7)

Second, senior leadership provided time and space for the partnership to evolve. To allow the partnership to evolve from a global idea to fit in Tanzania, Coca-Cola provided salary support for a full-time project manager from within the Coca-Cola system, and gave him a high degree of autonomy and flexibility to shape the Coca-Cola role of knowledge contributor. An executive from Coca-Cola commented on the freedom to innovate:

I think it’s been an incredibly positive aspect of the project that our leadership has given us the freedom to allow [the partnership] to develop into something. If we had been forced up front to define harder and clearer how exactly we were going to engage, I think it would’ve limited our innovation tremendously. (ID58)

#### Perceived benefits of the partnership

Although this process evaluation was not designed to quantify the impact of the partnership, participants identified a set of benefits that they believed they had realized through the collaboration (Table 4).

**Table 4:** Perceived benefits of the partnership.

Benefits to MSD	Enhanced collaboration and communication
	More proactive orientation in managing operations
	Greater attention to management of employee performance
Benefits to Coca-Cola	Strengthened knowledge transfer capability
	Enhanced job satisfaction

Participants from MSD described experiencing several benefits from the partnership, including a shift in MSD culture toward ‘a more of a private sector way of doing things’ (ID14). New cross-functional meetings, processes and tools introduced through the project: 1) facilitated greater collaboration and teamwork at MSD, 2) allowed MSD to adopt a more proactive orientation in managing operations, 3) improved system-wide communication, and 4) generated greater attention to the management of employee performance at MSD.

First, MSD staff experienced greater collaboration and communication within their organization. Participants shared how they gained a new appreciation for the interconnectedness of the supply chain, as well as the importance of alignment and accountability across all of the departments within MSD. Based on Coca-Cola’s planning processes, ADP facilitated the creation of structured, cross-functional bi-monthly meetings at MSD to facilitate integration across units. Several MSD staff reflected on how these meetings created a sense of teamwork and accountability as well as a formal structure for knowledge exchange in which employees would need to prepare, discuss, and then execute agreed upon actions. Planning had historically been relegated to “inventory analysts” who were located low within the organizational hierarchy. Through the partnership, the planning function was elevated to report directly to senior management and new roles of demand planner and supply planner were created to increase communication with end users and suppliers, respectively. As an employee of MSD commented:

I see now that we are more integrated in the units within ourselves, knowing that I cannot do it myself. I must make sure that all of the people involved in the whole supply chain are doing the right thing at the right time… So this is more of a private sector way of doing things. (ID14)

A staff member from MSD described:

The best success I saw was when we started having these bi-monthly meetings … cross-functional work where someone in the inventory department is able to point out when our stock outs are, and procurement would chime in and it’s like we can actually prevent this. If we didn’t have these meetings, we would have the stock out for a week. (ID9)

MSD staff also described a shift within MSD to become more proactive in managing operations, and to ensure information flow up the organization in order to preempt potential problems. In contrast to earlier, ‘reactive’ ways of working (ID30), MSD adopted some of Coca-Cola’s practices related to demand and supply planning. Reflecting on the changes that occurred throughout the project, a staff member from MSD described:

The big difference between the way we used to do it and the way we are training to do it now, is that our previous approach was very reactive. Things have already happened… Now the way we’re training our people now is to be proactive, to always be on top of what is happening on the field, filtering that information upwards, digesting it, and seeing potential things that would happen and take actions ahead of time. (ID30)

Finally, staff at MSD reported a greater recognition of the importance of monitoring employee performance. Through exposure to human resource processes at Coca-Cola, MSD sought to create job clarity and accountability among employees. Working with Coca-Cola and ADP, MSD designed a new performance management system (DRIVE) to allow employees to set concrete objectives that would be measured objectively on organizational values and rewarded based on improved performance. A staff member from the HR team of MSD explained the benefits of the new performance management system in this way:

So this [new performance management] system assures that every individual will work hard in order to get increase of his or her salaries. So we are sure that MSD will change and will grow, because every organization needs people who work hard to achieve objectives. So we will achieve more than what we expect. (ID49)

Participants from Coca-Cola also described having benefitted from the project in two ways: 1) developing stronger knowledge transfer capability and 2) enhanced job satisfaction. The process of learning how to effectively package and share its expertise with MSD prompted Coca-Cola to re-evaluate and improve upon some of its own practices, a sentiment shared by one employee who remarked “the best way to master something is to teach something” (ID56). Coca-Cola team members initially struggled to articulate the value of their expertise to MSD, ultimately recognizing the need to improve documentation of their processes and practices. A manager from Coca-Cola reflected:

After the last meeting [with MSD] we sat back and we said, we actually are so used to our processes that we don’t even know how to sell the benefits of what we do…so I asked [employees] to go and change all of our slides for every meeting that we have. I told them you have to go back to the drawing table and rethink about how to market the benefits of the processes that we have put in place…We launch all kinds of products… we think it’s normal to just keep doing it…And yet, we don’t talk about all the mess that we have gone through, the background and what we have saved and not saved the company from, and so we can go back and also begin thinking about how to package our reports differently. (ID53)

Staff from the Coca-Cola system also described enhanced job satisfaction associated with their participation in the project. The partnership provided an opportunity for employees of Coca-Cola to engage in corporate social responsibility efforts in a new, more emotionally meaningful way. An executive described the enhanced sense of meaning and motivation that staff derived from working towards a social good:

There’s been a motivational element, positive outcome, from a Coke system point of view. Even from those of us from a more corporate point of view, there’s no doubt there’s a sense of personal fulfillment of being able to contribute on something which has such a positive outcome of disease being prevented and lives being potentially saved. (ID58)

## Discussion

While the barriers to knowledge transfer across organizational boundaries are well described in theory [25, 27, 28], empirical evidence of barriers and facilitators of knowledge transfer across industry boundaries, particularly into global health, is limited. We found that MSD and Coca-Cola were able to overcome initial challenges, ultimately identifying and translating tools and processes to improve supply chain management. MSD staff also described a broader shift in organizational culture characterized by greater collaboration and communication, more proactive management of operations, and greater attention to performance management.

Our findings expand upon the knowledge transfer literature by providing novel descriptions of enabling factors that emerged in the experiences of MSD and Coca-Cola. First, our findings point to the importance of individual relationships in facilitating knowledge transfer. The social networks literature suggests that when people are connected on a personal level, they are better able to generate actionable knowledge [47, 48], and knowledge transfer across “strong” relationships is easier than across “weak” relationships [49]; however, transfer across weak ties is more likely to introduce novel knowledge [24]. Consistent with this literature, we find that investment of the time and attention needed to develop local relationships across boundaries was critical, and that shared activities such as joint warehouse visits were particularly valuable. This investment in understanding the partnership network and pursuing activities that convert individual relationships from weak to strong is likely to be particularly valuable in the development of partnerships that span vastly different organizational contexts, such as the differently calibrated incentives, authority structures, and access to resources across public and private domains [50]. Beyond partnership development, these personal connections may also have an impact on sustainability; for example, the enhanced job satisfaction identified by Coca-Cola may provide the intrinsic motivation for sustained knowledge transfer [31].

Second, prior research suggests that the recipient organization’s intent to learn is a key determinant of the extent to which knowledge is transferred [51], and that receptivity is an essential predictor of uptake of innovation in global health [52]. We found that MSD’s receptivity was shaped at both the political and the technical level. At the political level, the experience and comfort of MSD senior leadership with crossing social and organizational boundaries opened the door for continued refinement of the partnership model. At the technical level, we observed the that boundary spanners [53] were required to initiate novel connections, in both initial identification of knowledge to transfer (the supply chain consultant) as well translation from Coca-Cola to MSD during implementation (ADP consultants).

Given the complexities associated with emergent partnerships in global health [50], and acknowledging the possibility that the Coca-Cola to MSD boundary may be a pragmatic boundary [25], in which novelty generates different interests between actors that impede their ability to share and assess knowledge, is this partnership model a bridge too far to cross? The benefits perceived by MSD indicate that partners were able to transfer both explicit knowledge (i.e., bi-weekly planning processes) and tacit knowledge (i.e., greater emphasis on the management of employee performance) [23]. We also observed that the transfer of knowledge was promoted through the use of both operational tools that generated visible benefits, and ‘softer’ forms of transfer, including exposure to new behavioral norms [54]. Further study is required to determine whether these potentially deeper shifts in organizational culture can drive improved supply chain performance over the longer term, and whether they justify the required intensive investment in partnership development. Our findings should be interpreted in light of several limitations. First, our findings reflect participants’ experiences and perspectives during implementation; our study design did not include quantification of MSD’s performance, and hence we are unable to describe objective outcomes. Nevertheless, our qualitative approach was well suited for this process evaluation, where we sought to characterize knowledge transfer, a complex and dynamic phenomenon [55]. Based on the process evaluation described herein, a subsequent mixed-methods evaluation to quantify impact of this partnership across country settings is underway. Second, the single country setting may limit application to other settings; yet we provide a ‘thick description’ of partners, roles and context to enhance transferability of findings [44]. Third, there was potential for social desirability bias and fear of reprisal in participants’ responses [56]. We used several well-accepted techniques to minimize these influences, including establishing rapport, ensuring confidentiality, using trained interviewers and data collection strategies to encourage respondents to provide both positive and negative comments during interviews, encouraging respondents to share personal experiences in detail, and interviewing multiple staff members in each organization [35]. Fourth, it is important to differentiate between knowledge transfer (as described in this partnership) and knowledge translation (which would have required systematic evaluation of Coca-Cola’s business practices with the goal of translating the tested best practices into action) [21, 57, 58]. This partnership focused on the former: transfer of Coca-Cola’s experience and expertise in supply chain management to counterparts at MSD.

Finally, we feel it is imperative to anticipate concerns from some within the public health community about Coca-Cola’s interests in engaging in this initiative given the negative public health consequences of some of their products, especially in light of other industry examples of the use of CSR to gain public and political credibility [59]. This paper does not serve as an endorsement of Coca-Cola products or practices; we hope it promotes reflection and further scientific inquiry by serving as a useful example of a multinational corporation transferring its business expertise across industry boundaries with the goal of improving global health.

## Conclusions

Despite the desire of the global health community to learn from other industries, partnerships to transfer knowledge across boundaries have not been adequately explored in the literature. Linking theoretical constructs with practical experiences from the field, this paper highlights characteristics of a knowledge transfer partnership across industry boundaries that may be useful to others seeking to promote the transfer of knowledge to improve health.

## Abbreviations

ADP: Accenture Development Partnerships
CCK: Coca-Cola Kwanza
CSR: Corporate Social Responsibility
MSD: Medical Stores Department
TCCC: The Coca-Cola Company
USAID: United States Agency for International Development
